# Histopathological and behavioral evaluations of the effects of crocin, safranal and insulin on diabetic peripheral neuropathy in rats

**Published:** 2015

**Authors:** Amir Abbas Farshid, Esmaeal Tamaddonfard

**Affiliations:** 1 *Division of Pathology, Department of Pathobiology, Faculty of Veterinary Medicine, Urmia University, Urmia, Iran*; 2 *Division of Physiology, Department of Basic Sciences, Faculty of Veterinary Medicine, Urmia University, Urmia, Iran*

**Keywords:** *Crocin*, *Safranal*, *Insulin*, *Diabetic neuropathy*, *Rats*

## Abstract

**Objectives:**

Crocin and safranal, the major constituents of saffron, exert neuroprotective effects. In the present study, we investigated the effects of crocin and safranal (alone or in combination with insulin) on peripheral neuropathy in diabetic rats.

**Materials and Methods:**

Diabetes was induced by intraperitoneal (i.p.) injection of 60 mg/kg of streptozotocin (STZ) and confirmed by blood glucose level higher than 250 mg/dl. After confirmation of diabetes, crocin (30 mg/kg, i.p.), safranal (1 mg/kg, i.p.) (alone or in combination with insulin) and insulin (5 IU/kg, s.c.) were administered for eight weeks. Neuropathic pain was evaluated using acetone drop test. Histopathological changes of sciatic nerve were evaluated using light microscope. Blood glucose levels and sciatic nerve malondialdehyde (MDA) contents were also measured.

**Results:**

STZ caused cold allodynia, edema and degenerative changes of sciatic nerve, hyperglycemia and an elevation of sciatic nerve MDA levels. Crocin, safranal and insulin improved STZ-induced behavioral, histopathological and biochemical changes. Combined treatments produced more documented improving effects.

**Conclusion:**

The results of the present study showed neuroprotective effects of crocin, safranal and insulin in a rat model of diabetic neuropathy. In addition, crocin and safranal enhanced the neuroprotective effect of insulin. The neuroprotective effects of theses chemical compounds could be associated with their anti-hyperglycemic and antioxidant properties.

## Introduction

Peripheral neuropathy is a general term indicating a disorder of the peripheral nervous system (England and Asbury, 2004 ▶). The commonest cause of peripheral neuropathy is diabetes, and 30-90% of patients with diabetes have peripheral neuropathy (Callaghan et al., 2012 ▶). Although the pathogenesis of diabetic neuropathy is poorly understood, Zychowska et al. (2013) ▶ suggested that diabetic-induced sustained hyperglycemia is responsible for changes in the nerve tissue. In addition, reactive oxygen species (ROS) and reactive nitrogen species (RNS) production resulting from chronic hyperglycemia have important roles in pathogenesis of diabetic peripheral neuropathy (Premkumar and Pabbidi, 2013 ▶). These free radicals and some unidentified metabolic factors activate the nuclear enzyme poly (ADP-ribose) polymerase (PARP), which is a fundamental mechanism for complications observed in diabetic neuropathy (Zychowska et al., 2013 ▶). Diabetic peripheral neuropathy is characterized by pain, paraesthesia and sensory loss (Singh et al., 2014 ▶). 

Recent studies have suggested potent anti-diabetic effects for saffron (*Crocus sativus* L.) and its major constituents, crocin and safranal, in streptozotocin (STZ)-induced diabetic rats (Tamaddonfard et al., 2013b ▶; Rajaei et al., 2013 ▶; Samargandian et al., 2013 ▶; Asri-Rezaei et al., 2015 ▶; Altinoz et al., 2015 ▶). Also, using various nociceptive tests, pain-suppressing effects of crocin and safranal have been reported in rats (Tamaddonfard and Hamzeh-Gooshchi, 2010 ▶; Tamaddonfard et al., 2013c ▶; Karami et al., 2013 ▶; Tamaddonfard et al., 2014 ▶; Zhu and Yang, 2014 ▶; Erfanparast et al., 2015 ▶). In addition, crocin and safranal exerted neuroprotective effects on peripheral and central nervous systems (Tamaddonfard et al., 2013a ▶; Tamaddonfard et al., 2014 ▶; Sadeghnia et al., 2013 ▶; Alinejad et al., 2013 ▶). In STZ-induced type 1 diabetic rats, crocin and safranal exert potent antioxidant effects viadecreasing lipid peroxidation and improving catalase, superoxide dismutase activities (Rajaei et al., 2013 ▶; Samargandian et al., 2013 ▶). 

Based on the complex etiology of diabetic peripheral neuropathy, several treatment sincluding tricyclic antidepressants, selective serotonin reuptake inhibitors, antiepileptic drugs, narcotic agents and medicinal plants have been tested to halt its progression (Zychowska et al., 2013 ▶; Singh et al., 2014 ▶; El-Abhar and Schaalan, 2014 ▶; Javed et al., 2015 ▶). In this context, intraperitoneal (i.p.) injection of insulin (3 IU/kg) for 4 weeks improved electrophysiological and oxidative changes of sciatic nerve in STZ-induced diabetic peripheral neuropathy (Erken et al., 2015 ▶). In the present study, we investigated the effects of crocin, safranal (alone or in combination with insulin) and insulin on STZ-induced diabetic peripheral neuropathy by recording neuropathic pain signs, evaluation of sciatic nerve histological changes and measuring blood glucose and sciatic nerve malondialdehyde (MDA) levels in rats.

## Materials and Methods

**Animals**

In the present study, we used healthy adult male Wistar rats weighing 230-260 g. Animals were maintained in controlled laboratory conditions (12 h light/12 h dark cycles and ambient temperature 22 ± 0.5 ºC) with food and water *ad libitum*. All research and animal care procedures were approved by the Veterinary Ethics Committee of the Faculty of Veterinary Medicine of Urmia University and were performed in accordance with the National Institutes of Health Guide for Care and Use of Laboratory Animals.

**Chemical compounds**

Crocin was purchased from Fluka, Reidel-deHaen (Buchs SG, Schweiz). STZ and safranal (Kosher, purity of ≥ 88%) were purchased from Sigma-Aldrich Inc. (St Louis, MO, USA). Insulin was obtained from Exir Co. Pvt. Ltd. (Tehran, Iran). Crocin, STZ and insulin were dissolved in normal saline and safranal was dissolved in liquid paraffin (Tamaddonfard et al., 2013c ▶, 2014). All other chemicals were purchased from Merck Chemical Co. (Darmstadt, Germany).

**Experimental groups**

In the present study, 42 male Wistar rats were divided into seven groups of six rats. Group 1 (intact group) received citrate buffer followed by normal saline. Group 2 (STZ group) received STZ (60 mg/kg) followed by normal saline. Groups 3 (crocin group) received STZ followed by 30 mg/kg of crocin. Group 4 (safranal group) received STZ followed by 1 mg/kg of safranal. Group 5 (insulin group) received STZ followed by 5 IU/kg of insulin. Group 6 (crocin + insulin group) received STZ followed by crocin (30 mg/kg) plus insulin (5 IU/kg). Group 7 (safranal + insulin group) received STZ followed by safranal (1 mg/kg) plus insulin (5 IU/kg). Five days weekly i.p. injection of crocin and safranal and daily subcutaneous (s.c.) injection of insulin were done for eight weeks after confirmation of diabetes. The doses and injection routes of drugs used in the present study, were chosen according to previous studies in which crocin (7.5-30 and 15-60 mg/kg, i.p.), safranal (0.25-0.75 and 0.2-0.8 mg/kg, i.p.) and insulin (3, 4 and 5 IU/kg, s.c.) were used (Rajaei et al., 2013 ▶; Smargandian et al., 2013 ▶; Ranjitkumar et al., 2013 ▶; Wayhs et al., 2013 ▶; Tamaddonfard et al., 2013a ▶, 2014; Erken et al., 2015 ▶).

**Induction of diabetes**

Diabetes was induced in overnight-fasted rats by a single i.p. injection of 60 mg/kg of freshly prepared STZ. STZ was dissolved in sodium citrate buffer (0.1 M, pH 4.5). Hyperglycemia was confirmed by elevated blood glucose levels (higher than 250 mg/dl) in12-h fasted rats, determined on day 3 after injection of STZ, using a digital glucometer (Elegans, Germany). This method of measuring blood glucose level was also used on day 56, after confirmation of diabetes.

**Neuropathic pain test**

Cold allodynia which was used to evaluate neuropathic pain, was measured as the number of foot withdrawal responses after application of cold stimuli to the plantar surface of hind paw (Choi et al., 1994 ▶; Farshid et al., 2014 ▶; Nam et al., 2014 ▶; Tamaddonfard et al., 2014 ▶). One drop of 100% acetone was gently applied to the mid-plantar surface of the rat with a syringe connected to a thin polyethylene tube. A brisk foot withdrawal response after the spread of acetone over the plantar surface of the paw was considered as a sign of cold allodynia. The testing was repeated 10 times with an approximate interval 3-5 min between tests. Acetone drop test was repeated on days 10, 20, 30, 40 and 50 after confirmation of diabetes. The response frequency to acetone was expressed as a paw withdrawal frequency (PWF: number of paw withdrawals/number of trails × 100). 

**Tissue sampling**

On day 56, after confirmation of diabetes, the rats were euthanized and sciatic nerves were removed and divided into two segments. One segment was fixed in 10% buffer formal saline for histopathological evaluation; another segment was weighed and rinsed in ice-cold saline solution.

**Histopathological evaluation**

The formalin-fixed nerves routinely processed for paraffin embedding, thin (4-5 μm) sections were provided from nerves and stained with hematoxylin and eosin (H&E) for light microscopic observations. Evaluation of the nerve sections was based on the severity of the pathological changes including edema and degeneration. The following scores were given to lesions observed: 0 (none), 1 (mild), 2 (moderate) and 3 (severe) (Farshid et al., 2014 ▶).

**Biochemical assay**

Sciatic nerve segments were cut into small pieces, and then homogenized at 4°C in 2 ml of ice-cold saline with glass homogenizer. The resulting homogenate was passed through cellulose filter to remove impurities and divided into aliquots for biochemical analysis.

Lipid peroxidase was estimated by measurement of MDA levels spectrophotometrically in sciatic tissue homogenates according to method described by Ohkawa et al. (1979) ▶. Briefly, 0.1 ml of 20% trichloroacetic acid (TCA) and 1 ml of 0.8% of aqueous solution of thiobarbituric acid (TBA) were added to 0.1 ml of supernatant, then, mixed and incubated at 100 ºC for 80 min. After cooling on ice, samples were centrifuged at 4000 rpm for 10 min. After centrifugation, the absorbance of the organic layer was measured at 532 nm. The nmol MDA/g tissue was calculated using the plotted standard curve prepared from 1,1,3,3-tetraethoxypropane.

**Statistical analysis**

The results were analyzed using Graph Pad Prism version 5 (Graph Pad software Inc, US). Data obtained from acetone drop test was analyzed using two-way analysis of variance followed by Tukey’s test. Data obtained from histopathological changes, blood glucose levels and MDA of sciatic nerve contents were analyzed by one-way analysis of variance (ANOVA) followed by Tukey’s test. The values are expressed as mean ± SEM of six animals. P<0.05 was considered statistically significant.

## Results

**Figure 1:**
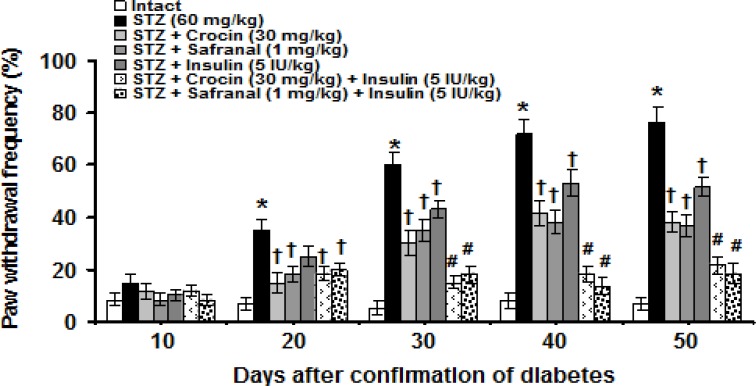
Effects of crocin, safranal (alone or in combination with insulin)and insulin on STZ-induced neuropathic pain. All values are expressed as mean ± SEM (n = 6). *p<0.001 in comparison with intact group.^†^p<0.01, ^#^p<0.05 in comparison with STZ group.STZ: streptozotocin

Plantar surface application of acetone in intact animals produced negligible paw responses. No significant responses to acetone were observed among experimental groups on day 10 after diabetes confirmation. Significant (p<0.001) differences in paw withdrawal frequency were observed between intact and STZ groups on days 20 (intact: 6.7 ± 2.4% vs STZ: 35 ± 4.5%) and 30 (intact: 5 ± 1.6% vs STZ: 60 ± 4.8%), 40 (intact: 8.3 ± 3% vs STZ: 71.7 ± 6.1%) and 50 (intact: 6.7 ± 2.3% vs STZ: 76.7 ± 6%) after confirmation of diabetes. Crocin (30 mg/kg) and safranal (1 mg/kg) significantly (p<0.01) suppressed neuropathic pain response on the above-mentioned days. Insulin (5 IU/kg) had no effects on day 20, whereas it significantly (p<0.01) reduced paw withdrawal frequency on days 30, 40 and 50. Crocin, safranal and insulin-treated groups showed no significant differences. In combined treatments, crocin (30 mg/kg) plus insulin (5 IU/kg) and safranal (1 mg/kg) plus insulin (5 IU/kg) produced more suppressive effect on STZ-induced cold allodynia (p<0.05, Figure 1). 

**Figure 2:**
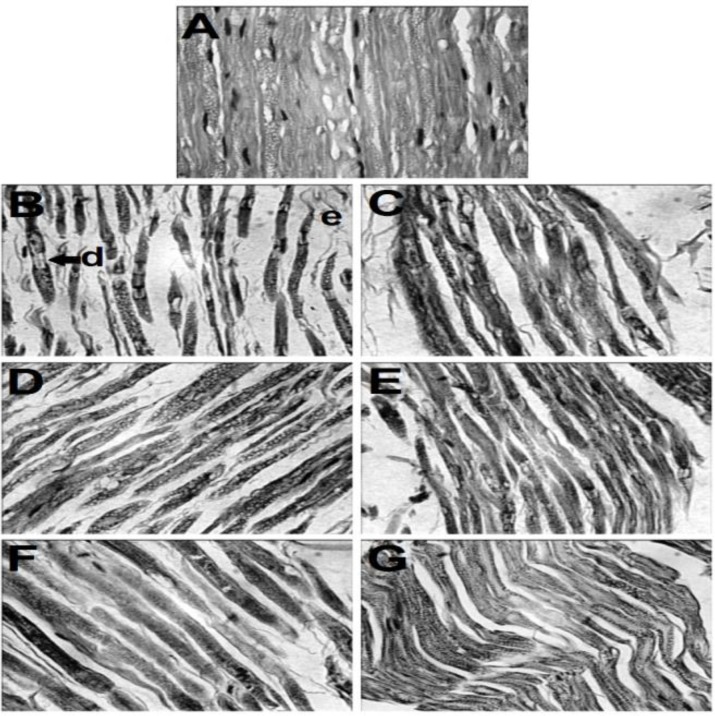
Histopathological analysis of rat sciatic nerve. (**A**): Normal architecture of sciatic nerve is evident in intact group. (**B**): Sever edema (e) and degenerative (d, arrow) changes are seen in STZ group. (**C**): Mild progression in pathological changes is seen in crocin (30 mg/kg)-treated diabetic rats. (**D**): Reduction of edema and degenerative changes is seen in safranal (1 mg/kg)-treated diabetic rats. (**E**): No degenerative changes are seen, but mild edema is still persisting in insulin (5 IU/kg)-treated diabetic rats. (**F**): A partial return of sciatic nerve to its normal structure is seen in crocin (30 mg/kg) plus insulin (5 IU/kg)-treated diabetic rats. (**G**): A complete return of sciatic nerve to its normal structure is evident in safranal (30 mg/kg) plus insulin (5 IU/kg)-treated diabetic rats (H&E**×**400

Table 1 and Figure 2A show the normal architecture of sciatic nerve tissue. STZ produced histopathological changes including edema and degeneration in sciatic nerve tissue (Table 1 and Figure 2B, p<0.001). 

**Table 1:** Effects of crocin, safranal (alone or in combination with insulin) and insulin on streptozotocin-induced sciatic nerve lesion severity score

**Groups **	**Sciatic nerve lesion severity score**
**Intact **	0.0 ± 0.0
**STZ (60 mg/kg) **	2.83 ± 0.17*
**STZ + Crocin (30 mg/kg) **	1.83 ± 0.34†
**STZ + Safranal (1 mg/kg) **	1.67 ± 0.26†
**STZ + Insulin (5 IU/kg) **	1.52 ± 0.22†
**STZ + Crocin (30 mg/kg) ** **+ Insulin (5 IU/kg) **	0.51 ± 0.24#
**STZ + Safranal (1 mg/kg) ** **+ Insulin (5 IU/kg)**	0.67 ± 0.21#

All values are expressed as mean ± SEM (n = 6).

* p<0.001 in comparison with intact group.

† p<0.05,

# p<0.001 in comparison with STZ group.

Crocin (30 mg/kg, Table 1 and Figure 2C), safranal (1 mg/kg, Table 1 and Figure 2D) and insulin (5 IU/kg, Table 1 and Figure 2E) significantly (p<0.05) but partially reduced STZ-induced changes. No significant differences were observed among crocin-, safranal- and insulin-treated groups. In combined treatments, crocin (30 mg/kg) plus insulin (5 IU/kg) and safranal (1 mg/kg) plus insulin (5 IU/kg) caused a significant (p<0.001) and complete reduction in histopathological effects (Table 1, Figures 2F and 2G).

**Figure 3:**
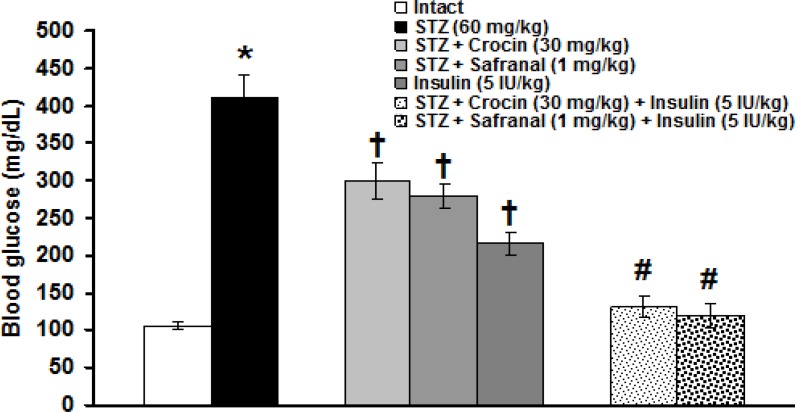
Effects of crocin, safranal (alone or in combination with insulin) and insulin on STZ-induced blood glucose level changes. All values are expressed as mean ± SEM (n = 6). *p<0.001 in comparison with intact group.†p<0.001, #p<0.05 in comparison with STZ group. STZ: streptozotocin

Blood glucose levels in intact group (105.7 ± 5.7 mg/dl) was significantly different from those of STZ (412 ± 29.9 mg/dL) group (p<0.001). Crocin (30 mg/kg), safranal (1 mg/kg) and insulin (5 IU/kg) significantly (p<0.001) decreased the increased levels of blood glucose. Blood lowering effects of insulin was significantly (p<0.05) more pronounced than those of crocin and safranal. No significant differences between crocin and safranal treated groups were observed. Crocin (30 mg/kg) and safranal (1 mg/kg) in combination with 5 IU/kg of insulin produced more documented anti-hyperglycemic effects (p<0.05, Figure 3). 

**Figure 4:**
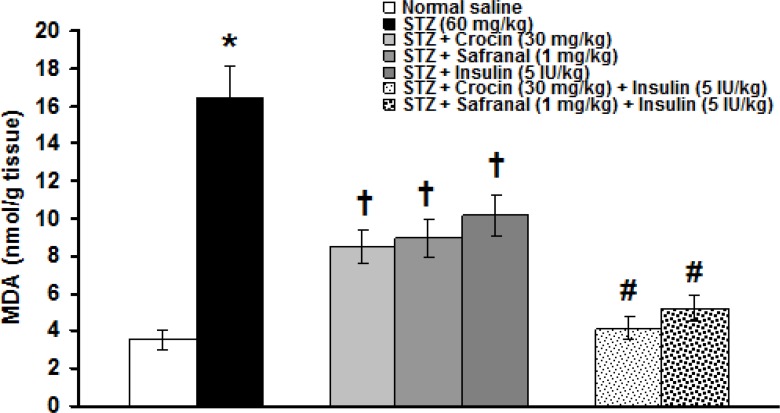
Effects of crocin, safranal (alone or in combination with insulin) and insulin on STZ-induced sciatic nerve MDA content changes. All values are expressed as mean ± SEM (n = 6). *p<0.05 in comparison with intact group.†p<0.05, #p<0.001 in comparison with STZ group. STZ: streptozotocin. MDA: malondialdehyde

A significant difference was observed between sciatic nerve tissue MDA levels in intact group and those of STZ group (3.51 ± 0.56 and 16.42 ± 2.14 nmol/g tissue, respectively, p<0.05). Combination treatments using crocin (30 mg/kg), safranal (1 mg/kg) withinsulin (5 IU/kg) significantly (p<0.05) decreased the increased levels of MDA induced by STZ. Crocin, safranal and insulin-treated groups showed no significant differences. The improving effects of combined treatments using crocin and safranal with insulin on sciatic nerve MDA levels were more documented (p <0.001, Figure 4).

## Discussion

In the present study, STZ caused cold allodynia, which began from day 20 after STZ and increased to the end of experiment. This confirms the finding of Nam et al., (2014) ▶ who reported that cold allodynia (using plantar surface application of acetone) is present from the second week after STZ (60 mg/kg) injection. In addition to cold allodynia, other sings of neuropathic pain including mechanical allodynia and heat hyperalgesia have been reported in STZ-induced diabetic neuropathy in rats (Nam et al., 2014 ▶; Bujalska-Zadrazny et al., 2015 ▶). Chronic hyperglycemia and the corresponding glucotoxicity induced by STZ are the main pathogenic mechanisms of diabetes and its complication (Wu and Yan, 2015 ▶). Histopathological changes of sciatic nerve tissue including fiber degeneration and edema observed in the present study are in agreements with the finding of Omran (2012) ▶ in which partial separation of myelinated nerve fibers, axonal atrophy, endoneural edema, fiber degeneration of sciatic nerve have been reported in STZ (65mg/kg, i.p.)-induced diabetic peripheral neuropathy in rats. In this context, STZ increased MDA and decreased superoxide dismutase (SOD) levels in sciatic nerve tissues of rats (Wu et al., 2012 ▶). It is believed that chronic hyperglycemia in diabetes is associated with damaged mitochondria, increased production of ROS, decreased nerve blood flow, reduced supply of trophic factors and slowed nerve conduction. With poor treatment, these effects can lead to degenerative abnormalities in peripheral nervous system (Tomlinson and Gardiner, 2008 ▶).

In the present study, crocin and safranal improved STZ-induced behavioral, histopathological and biochemical changes. Anti-hyperglycemic effects of crocin and safranal observed in the present study are consistent with other investigations (Tamaddonfard et al., 2013b ▶; Rajaei et al., 2013 ▶; Samargandian et al., 2013 ▶). There are no reports on the effects of crocin and safranal on neuropathic pain in STZ-induced diabetic neuropathy. In sciatic nerve-crush injury in rats, crocin and safranal attenuated cold and mechanical allodynia (Tamaddonfard et al., 2013a ▶; Tamaddonfard et al., 2014 ▶). In addition, in spinal cord injury model of neuropathic pain, an anti-nociceptive effect was reported for crocin (Karami et al., 2013 ▶). Carrageenan-induced inflammatory pain responses such as mechanical and cold allodynia were also suppressed by crocin and safranal (Tamaddonfard et al., 2013c). Using hind paw and orofacial formalin-induced pain tests, we showed anti-nociceptive properties of crocin (Tamaddonfard and Hamzeh-Gooshchi, 2010 ▶; Erfanparast 2015 ▶). Our results showed that crocin and safranal attenuated STZ-induced histopathological changes in sciatic nerve tissue. Although, there are no reports describing the effects of crocin and safranal on histopathological changes in STZ-induced diabetic neuropathy, in sciatic nerve-crush injury model, the inhibitory effects of crocin and safranal on Wallerian degeneration of sciatic nerve were reported (Tamaddonfard et al., 2013a ▶, Tamaddonfard et al., 2014 ▶). Our present results showed that crocin and safranal reduced sciatic nerve tissue levels of MDA in STZ-induced diabetic neuropathy. MDA is a reliable and commonly used biomarker for assessing lipid peroxidation. Lipid peroxidation is a well-established mechanism of cellular injury and is frequently used as an indicator of oxidative stress in cells and tissues (Moore and Roberts, 1998 ▶). It has been reported that crocin decreased MDA levels in kidney tissue in STZ-induced diabetic nephropathy in rats (Altinoz et al., 2015 ▶). Moreover, safranal decreased the elevated levels of MDA in rats heart tissue following isoproterenol-induced cardiomyopathy (Mehdizadeh et al., 2013). In sciatic nerve crush injury, crocin and safranal exerted potent antioxidant activities by reducing the blood levels of MDA (Tamaddonfard et al., 2013a ▶, 2014). It seems that anti-hyperglycemic and especially antioxidant activities of crocin and safranal contribute to their neuroprotective effects in STZ-induced diabetic neuropathy.

In this study, STZ-induced neuropathic pain signs, hyperglycemia, sciatic nerve histopathological and MDA level changes were improved following insulin treatment. Insulin is a pleiotropic hormone with many effects at the levels of cell, tissue and organism (Niswender, 2011 ▶; Mielke and Wang, 2011 ▶). STZ-induced changes such as sciatic nerve conduction velocity, compound action potential amplitude and total antioxidant status were improved by 3 IU/kg of insulin in rats (Erken et al., 2015 ▶). Although, Ranjithkumar et al., (2007) ▶ failed to show anti-thermal hyperalgesic effect for insulin (5 IU/kg for 4 weeks), chronic treatment with insulin (10 IU/kg for 4 weeks after 4 weeks induction of diabetes) attenuated thermal hyperalgesia and hot-plate latency in STZ-induced diabetic neuropathy in mice (Sharma et al., 2007 ▶). It seems that insulin with anti-hyperglicemic and antioxidant effects is involvedin induction of neuroprotection.

In the present study, STZ-induced behavioral, histopathological and biochemical changes improved more markedly after combined treatments using crocin and safranal with insulin. These can point that crocin and safranal may exert insulinomimetic effects. Although in the present study, the level of insulin was not measured, but according to our previous study, crocin at (15 and 30 mg/kg) administration for 30 days restored the blood levels of insulin in STZ-induced diabetic rats (Tamaddonfard et al., 2013b ▶; Asri-Rezaei et al., 2015 ▶). In addition, safranal activated insulin signaling in cultured myotubes and improved glucose tolerance in diabetic KK-Ay mice (Maeda et al., 2014 ▶). Current available therapies for diabetes and diabetic complications include insulin and various oral anti-diabetic agents. Many of these drugs have a number of serious adverse effects. Moreover, insulin treatment, particularly, if intensive, may be associated with emergency room visit and hospitalization due to hypoglycemic events. Therefore, the search for more effective and safer agents with combination therapy approaches is important (Sharma et al., 2007 ▶; Kubo et al., 2010 ▶; Patel et al., 2012 ▶). 

In conclusion, the results of the present study showed neuroprotective effects of crocin, safranal and insulin and pointed towards the beneficial effects of combination treatments with insulin in attenuating STZ-induced diabetic peripheral possibly through anti-nociceptive, anti-hyperglycemic, antioxidant and neuro-regeneration promoting mechanisms.

## References

[B1] Alinejad B, Ghorbani A, Sadeghnia HR (2013). Effects of combination of curcumin, linalool, rutin, safranal, and thymoquinone on glucose/serum deprivation-induced cell death. Avicenna J Phytomed.

[B2] Altinoz E, Oner Z, Elbe H, Cigremis Y, Turkoz Y (2015). Protective effects of saffron (its active constituent, crocin) on nephropathy in streptozotocin-induced diabetic rats. Hum Exp Toxicol.

[B3] Asri-Rezaei S, tamaddonfard E, Ghasemsoltani-Momtaz B, Erfanparast A, Gholamalipour S (2015). Effects of crocin and zinc chloride on blood levels of zinc and metabolic and oxidative parameters in streptozotocin-induced diabetic rats. Avicenna J Phytomed.

[B4] Bujalska-Zadrozny M, de Carde A, Pawlik K (2015). Influence of nitric oxide synthase or cyclooxygenase inhibitors on cannabinoids activity in streptozotocin-induced neuropathy. Pharm Rep.

[B5] Callaghan B, Cheng H, Stables C, Smith A, Felman F (2012). Diabetic neuropathy: manifestations and current treatments. Lancet Neurol.

[B6] Choi Y, Yoon YM, Na HS, Kim SH, Chung JM (1994). Behavioral signs of ongoing pain and cold allodynia in a rat model of neuropathic pain. Pain.

[B7] El-Abhar HS, Schaalan MF (2014). Phytotherapy in diabetes: review on potential mechanistic perspectives. World J Diabetes.

[B8] England JD, Asbury AK (2004). Peripheral neuropathy. Lancet.

[B9] Erfanparast A, Tamaddonfard E, Taati M, Dabbaghi M (2015). Effects of crocin and safranal, saffron constituents, on the formalin-induced orofacial pain in rats. Avicenna J Phytomed.

[B10] Erken HA, Genc O, Erken G, Ayada C, Gundagdu G, Dogan H (2015). Ozone partially prevents diabetic neuropathy in rats. Exp Clin Endocrinol Diabetes.

[B11] Farshid AA, Tamaddonfard E, Najafi S (2014). Effects of histidine and n-acetylcysteine on experimental lesions induced by doxorubicin in sciatic nerve of rats. Drug Chem Toxicol.

[B12] Javed S, Petropoulos IN, Alam U, Malik RA (2015). Treatment of painful diabetic neuropathy. Ther Adv Chronic Dis.

[B13] Karami M, Bathaie SZ, Tiraihi T, Habbibi-Rezaei M, Arabkheradmand J, Faghihzadeh S (2013). Crocin improved locomotor function and mechanical behavior in a rat model of contused spinal cord injury through decreasing calcitonic gene related peptide (CGRP). Phytomedicine.

[B14] Kubo S, Watada H, Kawamori R (2010). Combination therapy of miglitol and insulin in type 1 diabetes mellitus patients. Diabetes Investig.

[B15] Maeda A, kai K, Ishii M, Ishii T, Akagawa M (2014). Safranal, a novel protein tyrosine phosphatase 1B inhibitor, activates insulin signaling in C2C12 myotubes and improved glucose tolerance in diabetic KK-Ay mice. Mol Nutr Food res.

[B16] Mielke JG, Wang YT (2011). Insulin, synaptic function, and opportunities for neuroprotection. Prog Mol Biol Transl Sci.

[B17] Moore K, Roberts LJ (1998). Measurement of lipid peroxidation. Free Radical Res.

[B18] Nam JS, Cheong YS, Karm MH, Ahn HS, Sim JH, Kim JS, Choi SS, Leem JG (2014). Effects of nefopam on streptozotocin-induced diabetic neuropathic pain in rats. Korean J Pain.

[B19] Niswender KD (2011). Basal insulin: beyond glycemia. Postgrad Med.

[B20] Ohkawa H, Oishi N, Tagi K (1979). Assay for lipid peroxidase in animal tissues by thiobarbituric acid reaction. Anal Biochem.

[B21] Omran OM (2012). Histopathological study of evening primrose oil effects on experimental diabetic neuropathy. Ultrustruct Pathol.

[B22] Patel DK, Prasad SK, Kumar R, Hemalatha S (2012). An overview on antidiabetic medicinal plants having insulin mimetic property. Asian Pac J Trop Biomed.

[B23] Premkumar LS, Pabiddi RM (2013). Diabetic peripheral neuropathy: role of reactive oxygen and nitrogen species. Cell Biochem Biophys.

[B24] Rajaei Z, Hadjzadeh MA, Nemati H, Hosseini M, Ahmadi M, Shafiee S (2013). Antihyperglycemic and antioxidant activity of crocin in streptozotocin-induced diabetic rats. J Med Food.

[B25] Ranjitkumar R, Prathab Balaji S, Balaji B, Ramesh RV, Ramanathan M (2013). Standardized aqueous Tribulus terristri (Nerunjil) extract attenuates hyperalgesia in experimentally induced diabetic neuropathic pain model: role of oxidative stress and inflammatory mediators. Phytother Res.

[B26] Sadeghnia HR, Kamkar M, Assadpour E, Boroushaki MT, Ghorbani A (2013). Protective effect of safranal, a constituent of Crocus sativus, on quinolinic acid-induced oxidative damage in rat hippocampus. Iran J Basic Med Sci.

[B27] Samargandian S, Borji A, Delkhosh MB, Samini F (2013). Safranal treatment improves hyperglycemia, hyperlipidemia and oxidative stress in streptozotocin-induced diabetic rats. J Pharm Pharm Sci.

[B28] Sharma S, Chopra K, Kulkarni SK (2007). Effect of insulin and its combination with resveratrol or curcumin in attenuation of diabetic neuropathic pain: participation of nitric oxide and TNF-alpha. Phytother Res.

[B29] Singh R, Kishore L, Kaur N (2014). Diabetic peripheral neuropathy: current perspectives and future directions. Pharmacol Res.

[B30] Tamaddonfard E, Farshid AA, Ahmadian E, Hamidhoseyni A (2013a). Crocin enhanced functional recovery after sciatic nerve crush injury in rats. Iran J Basic Med Sci.

[B31] Tamaddonfard E, Farshid AA, Asri-Rezaee S, Javadi S, Khosravi V, Rahman B, Mirfakhraee Z (2013b). Crocin improved learning and memory impairments in streptozotocin-induced diabetic rats. Iran J Basic Med Sci.

[B32] Tamaddonfard E, Farshid AA, Eghdami K, Samadi F, Erfanparst A (2013c). Comparison of the effects of crocin, safranal and diclofenac on local inflammation and inflammatory pain responses induced by carrageenan in rats. Pharmacol Rep.

[B33] Tamaddonfard E, Farshid AA, Maroufi S, Kazemi-Shojaei S, Erfanparast A, Asri-Rezaei S, Taati M, Dabbaghi M, Escort M (2014). Effects of safranal, a constituent of saffron, and vitamin E on nerve function and histopathology following crush injury of sciatic nerve in rats. Phytomedicine.

[B34] Tamaddonfard E, Hamzeh-Gooshchi N (2010). Effect of crocin on the morphine-induced antinociception in the formalin test in rats. Phytother Res.

[B35] Tomlinson DR, Gardiner NJ (2008). Glucose neurotoxicity. Nat Rev Neurosci.

[B36] Wayhs CA, Tortato C, Mescka CP, Pasquali MA, Schnorr CE, Nin MS, Barros HM, Moreira JC, Vargas CR (2013). The association effect of insulin and clonazepam on oxidative stress in liver of an experimental animal model of diabetes and depression. Pharm Biol.

[B37] Wu YB, Shi LL, Wu YJ, Xu WH, Wang L, Ren MS (2012). Protective effect of glidazide on diabetic peripheral neuropathy through Drp-1 mediated oxidative stress and apoptosis. Neurosci Lett.

[B38] Wu J, Yan LJ (2015). Streptozotocin-induced type 1 diabetes in rodents as a model for studying mitochondrial mechanisms of diabetic β cell glucotoxicity. Diabetes Metab Syndr Obes.

[B39] Zhu KJ, Yang JS (2014). Anti-allodynia effects of safranal on neuropathic pain induced by spinal nerve ligation in rats. Int J Clin Exp Med.

[B40] Zychowska M, Rojewska E, Przewlocka B, Mika J (2013). Mechanisms and pharmacology of diabetic neuropathy-experimental and clinical studies. Pharmacol Rep.

